# Promoter-Bound p300 Complexes Facilitate Post-Mitotic Transmission of Transcriptional Memory

**DOI:** 10.1371/journal.pone.0099989

**Published:** 2014-06-19

**Authors:** Madeline M. Wong, Jung S. Byun, Maria Sacta, Qihuang Jin, SongJoon Baek, Kevin Gardner

**Affiliations:** 1 Genetics Branch, National Cancer Institute, Bethesda, Maryland, United States of America; 2 Laboratory of Endocrinology and Receptor Biology, National Institute of Diabetes and Digestive and Kidney Diseases, Bethesda, Maryland, United States of America; 3 Laboratory of Receptor Biology and Gene Expression, National Cancer Institute, Bethesda, Maryland, United States of America; Florida State University, United States of America

## Abstract

A central hallmark of epigenetic inheritance is the parental transmission of changes in patterns of gene expression to progeny without modification of DNA sequence. Although, the trans-generational conveyance of this molecular memory has been traditionally linked to covalent modification of histone and/or DNA, recent studies suggest a role for proteins that persist or remain bound within chromatin to “bookmark” specific loci for enhanced or potentiated responses in daughter cells immediately following cell division. In this report we describe a role for p300 in enabling gene bookmarking by pre-initiation complexes (PICs) containing RNA polymerase II (pol II), Mediator and TBP. Once formed these complexes require p300 to facilitate reacquisition of protein complex assemblies, chromatin modifications and long range chromatin interactions that enable post-mitotic transmission of transcriptional memory of prior environmental stimuli.

## Introduction

Mechanisms for the establishment of cellular memory of gene expression are necessary for the maintenance of cell fate decisions that establish lineages of specialized function in metazoan cells. Therefore, remembered patterns of gene expression must be faithfully transmitted and re-established in cellular progeny following cell division. To do this, information stored in a molecular form distinct from alterations in DNA sequence acquires the ability to: facilitate the maintenance of lineage specific patterns of gene expression; transmit memory of recent changes in the cellular environment; and establish early competence for gene expression upon mitotic exit [1], [2]. In general, these prerequisites are met by assemblies of sequence specific DNA binding protein and associated histone modifying and remodeling factors that must survive the massive disruption in chromatin structure and biochemistry that occurs during replication and condensation of mitotic chromatin in order to specify or re-establish genetic programs in daughter cells following mitosis. Specific “chromatin marking” mechanisms include histone modifications, deposition of histone variants, and the targeting by sequence-specific DNA binding transcription factors like HSF1, HSF2, RUNX2, GATA1, FOXA1 and TFIID [3]–[9]; which are thought to produce experimentally detectable changes in chromatin structure that persist throughout the cell cycle [10]. In addition, other factors involved in more general modes of chromatin regulation, including chromatin modifying factors like the histone methyl-transferase MLL and members of the BET family (Brd3, Brd4) have also been shown to have a role in transcriptional memory through the formation of diverse nuclear assemblies [11]–[13]. Collectively, these mechanisms have been referred to as molecular bookmarking [2], [14]–[16].

Prior reports of poised or preloaded RNA polymerase II (pol II) and p300/pol II complexes at genes in yeast, insect and mammalian cells [17]–[20] demonstrated that pol II containing complexes could be retained at gene promoters in the absence of a continuous stimulus. These observations suggested the intriguing possibility that promoter-bound pol II complexes might provide a “transcriptional memory” that could be transmitted to cellular progeny [20]. In this work we describe the observation that p300 forms stable assemblies with CREB, Mediator, TBP, Cohesin, Brd4 and pol II, that poise chromatin for transcriptional initiation and the re-acquisition of long range chromatin interactions to permit the post-mitotic, trans-generational transmission of transcriptional memory of prior gene activation expression events across multiple cycles of cell division. These findings illustrate that p300 facilitates the epigenetic transmission of inheritable gene expression programs and define and expand the central role for p300 in implementing and maintaining cell fate decisions during cellular differentiation.

## Results

### p300 mediates transgenerational transmission of prior transcriptional states

**Figure 1:**
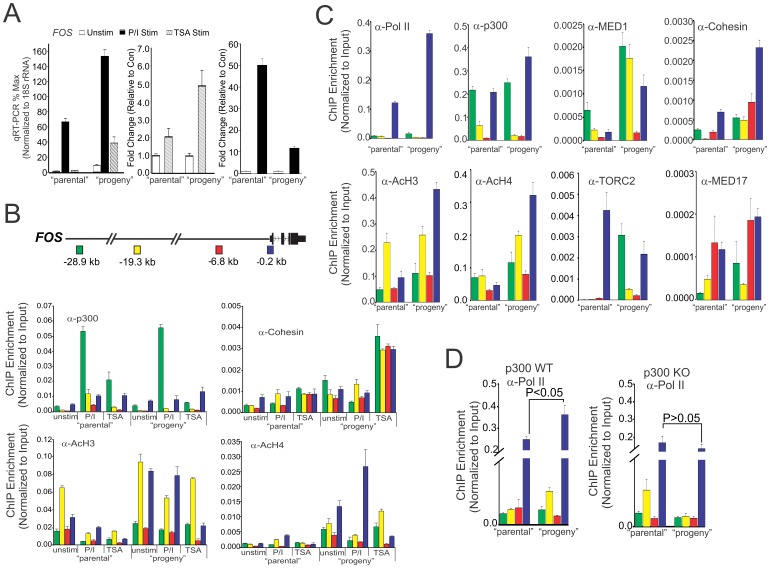
p300 facilitates parental trans-generational transmission of remembered states of potentiated transcriptional function. (A) Jurkat cells were treated with PMA and Ionomycin (P/I) for 1 h “parental”. Cells were then washed and allowed to rest for 40 h. qRT-PCR profile showing *FOS* expression in resting Jurkat cells (“parental”) or Jurkat cells pre-treated with P/I (mitogen pulsed) for 1 h, washed, allowed to rest for 40 h (“progeny”) and restimulated with P/I (1 h) and TSA (2 h). The fold induction upon TSA stimulation (middle) or P/I stimulation (right) are presented as relative level compared to the amount present in the unstimulated population. (B) Position dependent profile of p300, Cohesin, acetylated histone H3 (AcH3) and acetylated histone H4 (AcH4) at the *FOS* locus of Jurkat cells as determined by quantitative ChIP analysis. Error bars represent standard error of the mean from 2 biological replicates each determined in duplicate. Shown above is a schematic of the locations of enhancers (−28.9 kb & −19.3 kb), upstream (−6.8 kb) and promoter (−0.2 kb) at the *FOS* locus relative to TSS. (C) Position dependent profile at the *FOS* locus of Jurkat cells, relative to TSS, for indicated factors as determined by quantitative ChIP analysis. Error bars represent standard error of the mean from 2 biological replicates each determined in duplicate. (D) Position dependent profiles of pol II at the *FOS* locus in p300 WT and p300 KO in HCT 116 cells. Error bars represent standard error of the mean from 2 biological replicates, each determined in duplicate.

Previous studies have shown that following mitogen induction, p300 and pol II complexes show increased assembly at the promoters of immediate early genes like *FOS*, that persist for several hours in the absence of further stimulation [20]. Notably, the assembly of these complexes produced a potentiated state that enabled cells to respond more avidly to secondary challenges with weaker stimuli [20]. These observations suggested that persistently assembled p300/pol II complexes conveyed a transcriptional memory that potentiated more enhanced genetic responses upon subsequent environmental challenge. To assess the duration of this potentiated state, Jurkat T-cells were pulsed for 1 h with phorbol ester and ionomycin prior to mitogen washout and followed until 40 h later (Figure 1A and Figure S1A). After 40 h cells were harvested, stimulated a second time with either phorbol ester and ionomycin (P/I) or received heterologous stimulation with the histone deacetylase inhibitor trichostatin A (TSA). Transcriptional responses (*FOS* gene activation) were then compared to control cells similarly stimulated with P/I or TSA in the absence of pre-treatment (Figure 1A). In most mammalian cells, mitogen pulsing with P/I produces dramatic transient MAP kinase activation with subsequent short-lived increases in both the levels of phosphorylated extracellular signal regulated kinase (phospho-ERK) and phosphorylated cyclic-AMP response element binding protein (phospho-CREB), both major positive regulators of *FOS* transcription [21]. In Jurkat cells, both ERK and CREB phosphorylation are transient, each decaying to background levels within 4 h after stimulation with no evidence of activity at 40 h (Figure S1A). However at the level of transcription, as shown in Figure 1A, mitogen pre-treatment renders the cells more responsive to re-challenge with P/I or secondary activation with the much weaker, heterologous stimulant TSA (Figure 1A). This is not due to increased signaling through either phospho-CREB or phospho-ERK since re-stimulation through both pathways show reduced and or blunted mitogen induced phosphorylation (Figure S1B). Moreover, control and mitogen-pulsed cells showed nearly identical rates of cell division as demonstrate by carboxyfluorescein diacetate succinimidyl ester (CFSE) dye dilution assays [22], each passing through two cycles of cell division prior to re-stimulation at 40 h indicating that these changes in *FOS* expression are propagated across the cell cycle (Figure S1C).

ChIP analysis across the *FOS* locus reveals variable increase in the occupancy of p300, Cohesin, and the levels of acetylated histone H3 and H4 at the promoter and upstream regulatory regions (Figure 1B). Most notably p300 occupancy at the −28.9 kb upstream enhancers remains highly induced in parental and progeny cells in response to P/I stimulation, with significantly less accumulation in response to secondary stimulation with TSA. In contrast, accumulation of p300 at the promoters increases with P/I stimulation, remains elevated in progeny at the time of re-stimulation and rises to roughly equivalent levels following re-stimulation with P/I or TSA. In contrast Cohesin assembly shows variable increases in assembly at both enhancer and promoter regions in response to P/I and TSA stimulation of parental cells. This increase persists in progeny cells and becomes super-induced upon TSA stimulation (Figure 1B). Though the levels of histone H3 acetylation shows a slight decrease upon P/I stimulation of parental cells, this level is significantly elevated in the progeny cells independent of secondary P/I stimulation and shows a slight decrease with TSA stimulation (Figure 1B). This influence on histone H3 acetylation is in contrast to the effect of P/I on histone H4 acetylation in progeny, which shows dramatic increases at the promoter that potentiated with P/I. Secondary stimulation of progeny with TSA has variable influences with a predominant lost of levels at the promoter (Figure 1B). Such dynamic profiles in histone acetylation, p300 and Cohesion occupancy indicate a complicated interplay between p300 and a multiple of chromatin associated factors and binding events that is persistently transmitted at varying levels to progeny to influence subsequent complex assembly and transcriptional output.

Consistent with changes in the stimulated profiles of progeny cells, a closer comparison of complexes that remain assembled at the *FOS* locus, 40 h after the initial mitogen pulse (P/I washout), show significantly higher levels of pol II, p300, MED1, Cohesin, and histone H3/H4 acetylation at the *FOS* promoter and upstream enhancer elements than untreated controls (Figure 1C). In contrast, other transcriptional components involved in stimulus evoked activation including, the CREB-specific coactivator, TORC2 [23], and the MED17 co-regulator subunit, show either little change or decreased occupancy at the *FOS* promoter in progeny cells (Figure 1C). p300 is required for this “memory” function as mitogen-pulsed cells deficient in p300 do not show enhanced retention of pol II assemblies at the *FOS* promoter when examined 40 h post stimulation (Figure 1D).

### p300-containing PIC assemblies are retained at the *FOS* promoter and enhancers

**Figure 2:**
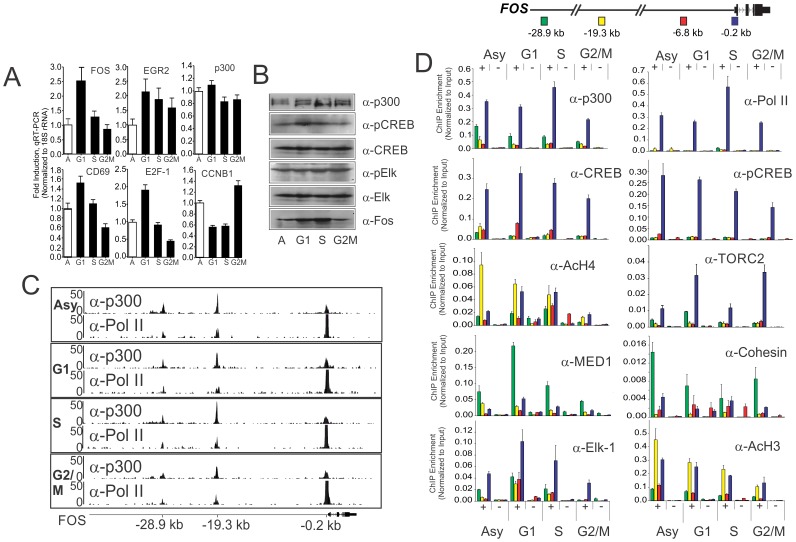
A pre-initiation complex containing p300 remains assembled at the *FOS* promoter and distal enhancers throughout the cell cycle. Jurkat cells were elutriated to obtain populations of cells at different stages of the cell cycle. (A) qRT-PCR profile showing expression of immediate early genes peaks at the G1 phase of the cell cycle. The fold induction present at each phase (G1, S and G2/M) is presented as relative level compared to the amount present at the asynchronous phase (A) Error bars represent standard error of the mean from 4 biological replicates each determined in triplicate. (B) Western blots showing protein levels of p300, total and phospho-CREB, total and phospho-Elk and FOS at different stages of the cell cycle. Shown is 1 of 3 independent biological replicate of elutriations. (C) ChIP-Seq profiles of the binding of p300 and pol II at the *FOS* promoter and distal enhancers. Shown is 1 of 2 independent biological replicate of elutriations. (D) Position dependent profile at the *FOS* locus for indicated antibodies (+) and no antibody control (−) as indicated determined by quantitative ChIP analysis throughout the cell cycle. Error bars represent standard error of the mean from 3 or 4 biological replicates each determined in duplicate. Above is shown a schematic of the locations of enhancers (−28.9 kb & −19.3 kb), upstream (−6.8 kb) and promoter (−0.2 kb) at the *FOS* locus relative to TSS as indicated.

The persistent accumulation of p300/pol II and histone acetylation at the *FOS* promoter during at least two cycles of cell division suggests that these complexes must remain assembled at the *FOS* promoter throughout the cell cycle. To test this possibility, Jurkat T-cells were purified in different phases of the cell cycle by centrifugal elutriation [24]. This procedure isolates highly enriched fractions of cells in G1, S, and G2/M phase of the cycle by direct fractionation according to size and buoyant density in the absence of the confounding influences of cellular stress caused by the mitotic poisons traditionally used to synchronize cellular populations *in vitro* (Figure S2). Analysis of elutriated cells in Figure 2A reveals that there is a significant increase in the expression of the immediate early genes *FOS*, *EGR2* and *CD69* upon entry into G1. In contrast, p300 transcription remains constant across the cell cycle. Phase specific expression of *CCNB1* (G2/M), and *E2F1* (G1) are provided as control markers for the cell cycle fractionation [25].

The mechanism of transcriptional regulation at the *FOS* promoter has been studied extensively and has become a well-established paradigm for understanding the control of immediate early gene transcription [26]. Dynamic histone acetylation/deacetylation occurs at the *FOS* promoter where the histone acetyl-transferase (HAT) activity of p300 plays a significant role [26]–[28]. Key elements in p300 recruitment include the serum-response-factor (SRF) and members of the ETS family of transcription factors [26], [27], [29]. During induction of mitogen activated protein kinase (MAPK) cascades, phospho-ERK causes conversion of the ETS family member Elk1 to phospho-Elk1. Phospho-Elk1 then undergoes conformational changes that enhances its interaction with p300, which in turn also allosterically increases the intrinsic HAT activity of p300, thus contributing further to the dynamic chromatin remodeling through histone acetylation [29]. An important additional factor in p300 recruitment to the *FOS* promoter is CREB which is constitutively bound to the *FOS* promoter, but increases its interaction with p300 significantly following mitogen induced phosphorylation [30], [31]. Thus, the *FOS* promoter is controlled by diverse multivalent interactions involving both pol II, Mediator and multiple sequence-specific DNA binding factors that enforce and enhance p300 interactions with the promoter.

Consistent with their requirement in *FOS* activation, western blot analysis confirms that p300, Elk, phospho-Elk and total CREB levels are readily detected throughout the cell cycle though essentially unchanged, while levels of phospho-CREB peak in G1 and S phases (Figure 2B). The peak of FOS protein corresponds closely with the phospho-CREB peak. ChIP-seq profile of pol II and p300 occupancy across the *FOS* locus in cell cycle fractionated Jurkat T-cells confirms the persistence of p300 at the *FOS* promoter and upstream enhancer regions [32] regions (Figure 2C). Consistent with this observation, ChIP validation demonstrates that assemblies containing pol II, p300, CREB, phospho-CREB, Mediator, Cohesin, the CREB co-activator, TORC2, Elk and acetylated histone H3/H4 remain elevated at the *FOS* promoter throughout G1, S, and G2/M phases of the cell cycle with notable decreases in occupancy at enhancer regions as cells progress to G2/M (Figures 2D). Finally, as anticipated, histone H3 density is the lowest, activating histone H3 tri-methylation at lysine 4 (H3K4Me3) is the highest, and repressive histone H3 tri-methylation at lysine 27 (H3K27Me3) is the lowest at the *FOS* promoter, consistent with local persistence of portions of the PIC assembly throughout the cell cycle (Figure S3). Together, these data suggest that multiple components of the pre-initiation complex (PIC), in conjunction with Mediator and Cohesin, remain variably assembled at the *FOS* promoter and distal enhancers throughout the cell cycle, to potentiate both early transcriptional induction and the re-acquisition of 3 dimensional chromatin structure. These assemblies are highly gene specific since both p300 and pol II are absent from the *IL2* promoter in mitotic chromatin even though IL2 is competent for expression in Jurkat T-cells (Figure S4), suggesting that such assemblies could be specific for rapidly inducible genes.

### p300-containing PIC assemblies are retained in mitotic chromatin

**Figure 3:**
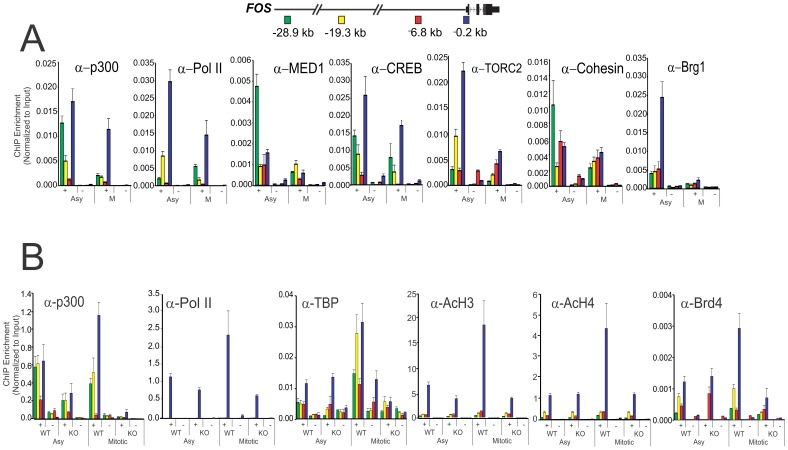
p300 is required for retention of enhancer and promoter bound PIC components in mitotic cells. (A) Position dependent profile at the *FOS* relative to TSS of indicated factors (+) and no antibody control (−) determined by quantitative ChIP analysis in asynchronous (Asy) versus mitotic (M) Jurkat cells. Error bars represent standard error of the mean from 3 biological replicates each determined in duplicate. Jurkat cells were treated with nocodazole (400 ng/ml) for 24 h to obtain metaphase (M-phase) population. (B) Position dependent profile at the *FOS* locus relative to TSS of indicated factors (+) and no antibody control (−) determined by quantitative ChIP analysis in asynchronous (Asy) versus mitotic (M) p300 WT and p300 KO in HCT 116 cells. Error bars represent standard error of the mean from 2 biological replicates each determined in duplicate. A schematic of the locations of enhancers (−28.9 kb & −19.3 kb), upstream (−6.8 kb) and promoter (−0.2 kb) at the *FOS* locus relative to TSS is shown above.

It has been well established that most of the transcriptional apparatus and many transcriptional regulators are displaced from the nucleus and chromatin during mitosis [15], [16], [36]. Some studies suggest that the majority of p300 is excluded from the nucleus [37]. However, other studies contradict this finding [5], [38]. In all likelihood, this partitioning will be cell-specific [15], as has been the case for Brd4 [11], [12]. Microscopic analysis of mitotic cells in metaphase shows that, in Jurkat, the vast majority of p300 is excluded from chromatin, however there are multiple scattered regions in which p300 chromatin retention is morphologically detectable (Figure S5). Though centrifugal elutriation produces highly enriched populations of cells in G1, S and G2/M; the levels of mitotic cells in G2/M is less than 20%. Therefore, in order to demonstrate that PIC assemblies remain at the *FOS* promoter in mitotic chromatin, Jurkat cells were blocked with the mitotic inhibitor nocodazole, which captures nearly 88% of cells in M-phase (Figure S6). Analysis of these cells by ChIP reveals persistently high levels of pol II, p300, CREB and to a lesser extent, Mediator, TORC2 and Cohesin at the *FOS* promoter (Figure 3A). Allow there are more dramatic losses at the enhancer regions, the significant selective enrichment and partial retention of p300, pol II, Mediator, Cohesin and CREB at the upstream enhancer regions, suggest that remnants of the 3 dimensional chromatin-associated structures, in complex with components of the transcriptional apparatus, remain assembled at both promoter and distal regulatory regions. Though reduced an average of 40% below the levels of untreated cells, this degree of binding is much higher than would be estimated from a 9% contamination of non-mitotic cells in nocodazole treated preparations. In contrast, although Brg1 is enriched at the *FOS* promoter in quiescent cells, it is nearly completely displaced during mitosis (Figure 3A). These findings demonstrate that several components of the transcriptional apparatus survive the massive condensation of chromatin during mitosis so that they may seed and reconstitute the various functional complexes and chromatin conformations necessary for effective gene expression following exit from mitosis.

To assess the requirement for p300 in the assembly of the PIC complex throughout the cell cycle, the promoter and enhancer occupancy of pol II, TBP and histone H3/H4 acetylation were compared in cells in which expression of a functional p300 had been deleted by homologous recombination [39]. Control HCT116 (p300 WT) and p300 deleted (p300 KO) cells were compared for the assembly of PIC components in mitotic cells (Figure 3B). p300 KO cells show significantly decreased assembly of pol II, TBP and histone H3 and H4 acetylation. In contrast, the bromodomain containing nuclear protein Brd4, which has been shown to partition diffusely onto mitotic chromatin and associate with numerous genes that are programmed to be expressed immediately upon exit from mitosis [12], shows elevated binding in mitotic chromatin that is lost after p300 depletion (Figure 3B).

### p300 facilitates rapid reconstitution of long-range chromatin interactions

**Figure 4:**
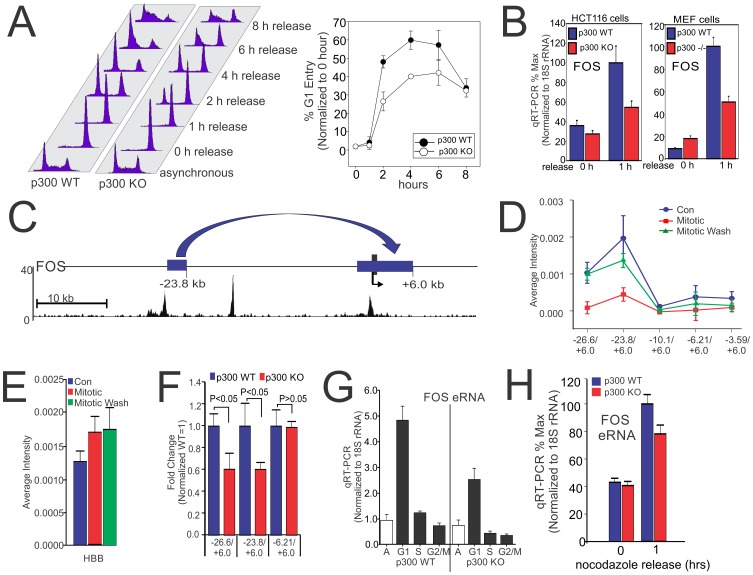
p300 is required for G1 re-entry and re-establishment of long range chromatin interaction at the *FOS* locus. (A) p300 WT and p300 KO HCT116 cells were treated as in Figure 3B, and released after washing. Cell cycle analysis by flow cytometric (FACS) for DNA content (Propidium Iodide staining) of cell before (Asynchronous) and after release from nocodazole treatment (16 h). Graphical representation of percentage of G1 cells is shown. Error bars represent standard error of the mean from 2 biological replicates. (B) qRT-PCR profile showing expression of *FOS* in the respective cell lines upon nocodazole treatment and release. Error bars represent standard error of the mean from 2 biological replicates. (C) A schematic of the locations of Chromosome Conformation Capture (3C) sites at the *FOS* locus relative to TSS with p300 localization as determined by ChIP-Seq. Jurkat cells were treated as in Figure 3A and washed and collected after 2 h. 3C assay was carried out and average intensity of PCR bands of respective primers of the *FOS* locus [Chr 14: 74822063 (+6.0 kb), 74788795 (−26.6 kb), 74791374 (−23.8 kb), 74805069 (−10.1 kb), 74808985 (−6.2 kb) and 74811601 (−3.59 kb)] (D) and *HBB* locus [Chr 11: 5209027 and 5199783] (E) were quantified. Error bars represent standard error of the mean from 3 biological replicates. (F) Comparison of 3C sites of the *FOS* locus between p300 WT and p300 KO in HEK293 cells. Error bars represent standard error of the mean from 5 biological replicates. (G) qRT-PCR profile showing expression of *FOS* enhancer RNA (eRNA) across the cell cycle in p300 WT and p300 KO cells. Error bars represent standard error of the mean from 2 biological replicates each determined in triplicate. (H) qRT-PCR profile showing expression of *FOS* eRNA expression in p300 WT or p300 depleted cells upon nocodazole treatment and released. Error bars represent standard error of the mean from 2 biological replicates determined in triplicate.

To profile the influence of p300 on early gene expression and post-mitotic cell cycle progression, p300 WT and p300 KO cells were compared for their rate of cell cycle re-entry following release from blockade by nocodazole washout (Figure 4). The percent cellular distribution in G1, S and G2/M at 0, 1, 2, 4, 6, and 8 h following release was profiled by fluorescence activated cell sorting analysis (FACS) of propidium iodide stained cells (Figure 4A). Direct comparison of the cell cycle progression profiles of p300 WT versus p300 KO cells reveals a significant decrease in the post-mitotic progression into G1 in the p300 KO cells (Figure 4A). This is consistent with prior observations that early post-mitotic expression of *FOS* is required for cell cycle progression [40]. Similar delays in cell cycle entry are obtained by blocking p300/CBP recruitment to the *FOS* promoter using a dominant negative CREB construct [41] or global transcriptional inhibition by actinomycin D (Figure S7). Finally, the requirement for p300 in the post-mitotic expression of *FOS* is demonstrated further by the observation that both HCT116 cells and mouse embryonic fibroblasts (MEF) depleted of p300 by homologous recombination, show delayed post-mitotic *FOS* expression (Figure 4B).

To test whether the *FOS* promoter and the putative upstream enhancer regions form 3 dimensional structures through chromatin looping, chromatin conformation capture assays (3C) were performed (Figures 4C–F). Control cells, nocodazole-blocked mitotic cells, and cells 2 h after nocodazole release (Mitotic wash) were quantitatively compared for interaction between the *FOS* promoter region and its −23.8 kb upstream regulatory region (Figures 4C–D). As shown in Figure 4D, interactions between the upstream −23.8 kb region and the *FOS* promoter region was readily detected in control cells, but was completely lost in mitotic chromatin. However, 2 h following nocodazole washout and mitotic exit the chromatin looping was quickly re-established (Figure 4D). In contrast, the hemoglobin locus, which is silent in Jurkat T-cells, shows little difference in interactions in control, mitotic or cells following mitotic exit. The slight increase in detectable interactions instead of decrease during mitosis, most likely reflects the influence of chromatin condensation (Figure 4E).

To examine the p300 requirement for this chromatin confirmation, the 3C assay was performed using either control HEK293 cells, or HEK293 cells depleted of p300 (Figure S8). The interaction between upstream −23.8 kb region and the *FOS* promoter region was significantly disrupted in p300 knock out cells (Figure 4F) while very little change is seen in the control interaction regions between −6.21 and +6.0 kb relative to the *FOS* start of transcription (Figure 4F). Similarly, expression of enhancer associated RNA (eRNA) [42] is significantly impaired in p300 depleted cells compared to WT Jurkat cells (Figure 4G). Similar though less significant differences in eRNA are seen following nocodazole washout in HCT116 cells depleted of p300 (Figure 4H).

### p300 enhances post-mitotic loading of Brd4 and Cohesin

**Figure 5:**
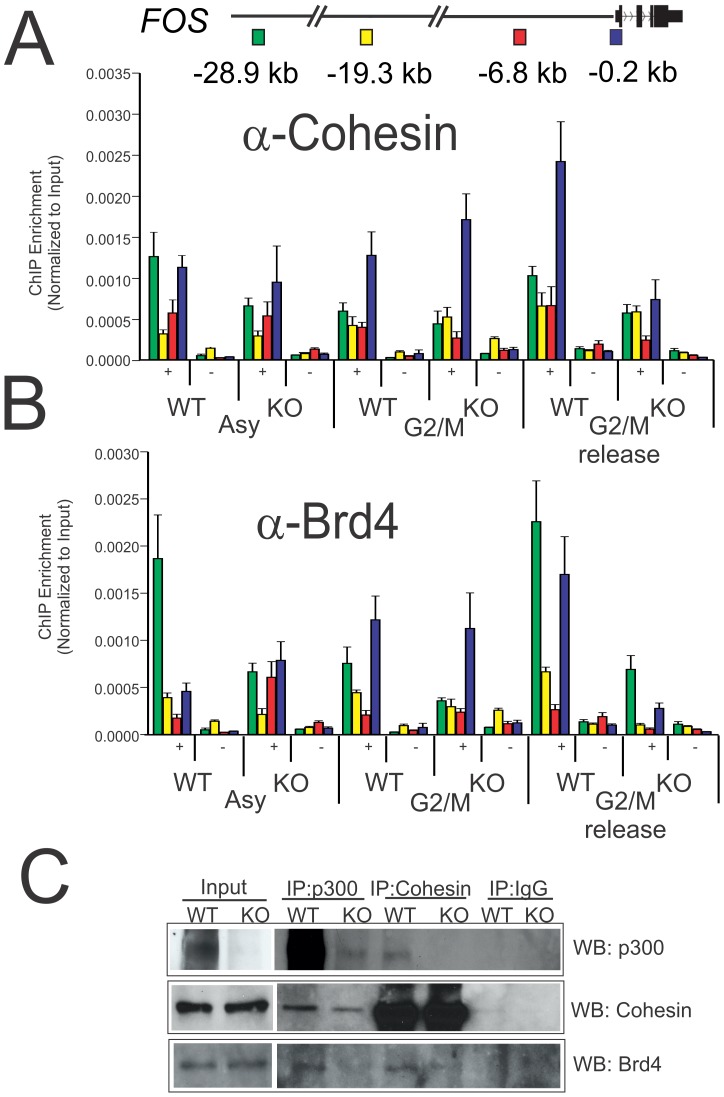
p300 is required for post-mitotic recruitment of Cohesin and Brd4. Jurkat p300 WT and p300 KO cells were elutriated to obtain cells of G2/M population and allowed to progress for 20 min to the G1 phase of the cell cycle. A schematic of the locations of enhancers (−28.9 kb & −19.3 kb), upstream (−6.8 kb) and promoter (−0.2 kb) at the *FOS* locus relative to TSS as indicated. Position dependent profile at the *FOS* locus for (A) Cohesin and (B) Brd4 antibodies (+) and no antibody control (−) as indicated determined by quantitative ChIP analysis. Error bars represent standard error of the mean from 2 biological replicates each determined in duplicate. (C) ChIP-western showing p300 dependency for Cohesin and Brd4 interaction. Shown is 1 of 2 independent biological replicates.

Recent findings indicate a widespread role for the Cohesin complex in the assembly of long range chromatin interactions between gene promoters and enhancers [33]–[35], [43]. Available evidence suggests that, similar to its role in linking sister chromatids, Cohesin can also function to regulate transcription by physically tethering promoter and enhancer regions [33]. The transcriptional coactivator, Mediator, is capable of forming complexes with Cohesin and provides a means of loading the Cohesin ring at promoter/enhancer pairs. Mediator is also a well-known binding partner for p300 [44], [45], and p300 is a well-established marker for distal enhancers [46], so it is very likely that p300 interactions may also have a role in loading Cohesin complexes at promoter-enhancer pairs during the formation of chromatin loops. To determine the requirement for p300 in post-mitotic loading of Cohesin at the *FOS* promoter and enhancer pairs, the occupancy of Cohesin in control and p300 depleted G2/M isolated Jurkat cells was compared following 2 h release into G1. Loss of p300 resulted in a significant decrease in recovery of Cohesin binding at the *FOS* promoter and upstream enhancer regions following mitotic exit and entry into G1 (Figure 5A).

Brd4 plays a significant role in regulating transcriptional elongation by binding to and recruiting transcriptional elongation factors to active genes [12], [47], [48]. Dey *et al.* have recently shown that this function for Brd4 is required for post-mitotic recovery of genes that are expressed early following mitosis and remains bound to chromatin to mark genes for early expression through this mechanism [12]. This observation has been expanded to show that Brd4 can also mark genes that were transcriptionally active just prior to the onset of mitosis, so that they may show potentiated expression following mitotic exit [11]. We have previously shown that p300 deficient HCT116 cells show significant loss of Brd4 from mitotic chromatin (Figure 3B). To test whether or not p300 is required for post-mitotic recruitment of Brd4 during the recovery of early gene expression upon entry into G1, Brd4 occupancy at the *FOS* promoter and enhancer regions was compared in wild type and p300 depleted G2/M purified Jurkat cells (Figure 5B). As shown in Figure 5B, loss of p300 results in significant deficiency in Brd4 recruitment following re-entry into G1. This observation is consistent with the decreased recovery of *FOS* transcription following mitotic release (Figure 4B). Finally, immuno-precipitation assays show that both Cohesin and Brd4 form detectable complexes with p300 in HEK293 cells (Figure 5C). Both of these interactions, including an interaction between Brd4 and Cohesin are reduced in p300 depleted cells (Figure 5C). These finding suggests that p300 coordinates many interactions at the *FOS* promoter and enhancer to facilitate the formation of diverse components of gene bookmarking complexes.

## Discussion

**Figure 6:**
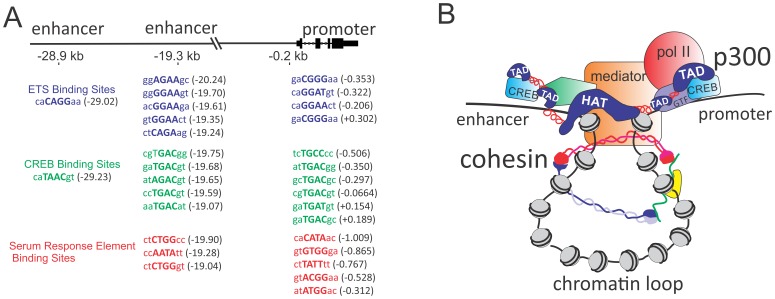
Role of p300 in stabilizing long-range promoter enhancer interactions. (A) Schematic table showing clusters of ETS, CREB, and SRE binding sites shared across enhancer and promoter elements at the *FOS* locus. (B) Schematic representation of possible interaction between p300, Mediator, Cohesin and bound general and sequence specific transcription factors.

p300 is a highly versatile adaptor protein with many multivalent interactions. p300 and its paralog CBP have 4 transactivation domains (TADs) and multiple other protein interaction interfaces interconnected by more flexible unstructured intervening regions that add significant adaptability to its structure [49]–[51]. In addition to the TADs, p300/CBP also contains multiple chromatin interacting domains including the HAT domain, an adjacent Bromo domain that, like Brd4, recognizes acetylated histone tails, and a cysteine-histidine rich plant homeodomain (PHD) also thought to mediate interactions with histone [51]. The TADs of p300 mediate protein interactions with a variety of protein and protein complexes including DNA binding transcription factors, general transcription factors and transcriptional coactivators. Most promoters and enhancers have multiple different factor binding sites in a variety of different configurations often repeated at associated enhancers and promoters [52] (Figure 6A). Thus, p300 can provide a highly adaptive and flexible interface not only with the potential of linking various internal promoter and enhancer interactions, but also the ability to form interactions across approximated promoter/enhancer interfaces produced by chromatin looping. These potential configurations indicate that p300 could play a diverse role in forming and stabilizing long range chromatin interactions via direct physical interactions distinct from its HAT activity, including the bridging of interactions between acetylated histone and transcription factor TADs bound at either promoter or enhancer regions or both (Figure 6B). The finding that p300 can form complexes with Cohesin (Figure 5) combined with its known interactions with Mediator [44], [45] makes this role highly plausible.

The p300 requirement for post-mitotic recruitment of Brd4 may arise from multiple mechanism, including increased chromatin accessibility, increased Histone 4 Lys 5 acetylation [53] (a preferred histone modification for Brd4 interactions), and the ability of p300 to recruit P-TEFb and other elongation factors [20], [54], [55]. Thus, the recently reported role for H4 Lys 5 acetylation (H4K5ac) in the post-mitotic reactivation of transcription through Brd4 [11] is likely to involve p300. Interestingly, the recently reported histone marks preferentially deposited by p300 and CBP including H3 Lys 27 acetylation (H3K27ac) and H3 Lys 18 acetylation (H3K18ac) do not appear to depend on p300 alone [56]. Similarly, histone marks and variants that have been commonly associated with enhancers or active promoters including the H3 Lys 4 mono-methylation (H3K4Me1), and H2A.Z deposition [57], do not appear to be altered by p300 depletion (Figures S8–S9). It is possible that these modifications may be sufficiently compensated by the activity of the p300 paralog CBP. The double p300/CBP knockout cells grow very slowly therefore were not experimentally accessible for cell cycle based studies because they could not be grown in sufficient numbers for extensive molecular and biochemical analysis [56].

It has been known for nearly 50 years that there is a general cessation of transcription upon entry into mitosis [58]. This is accompanied by the bulk displacement of transcriptional activators, general transcription factors, chromatin modifying factors, pol II, elongation factors and other components of the molecular machinery that participate in the biosynthesis and export of messenger RNA [4], [36], [37], [59]–[61]. The period of repressed transcription begins late in prophase and ends in late telophase with a general increase in the accessibility of chromatin and the appearance of early transcription followed by an ordered re-entrance of the expelled transcriptional machinery into the newly formed nuclei of daughter cells [60], [62]. During this period, old genetic programs are altered or re-established and new fate decisions initiated. Results from this study suggest a role for p300 in providing a means through which this genetic program can be dynamically re-wired and transmitted to cellular progeny in rapid response to environmental changes in both health and disease.

Several recent studies have begun to develop a clearer picture of the sequence of events that unfold to determine how lineage specific gene regulatory programs are maintained or re-directed following cell division [1], [2], [8], [9], [14], [15], [63], [64]. Though these bookmarking mechanisms are clearly distinguishable and include the association of general and sequence specific DNA binding factors and the marking or tagging of defined chromatin locales by the placement of histone variants and/or covalent chromatin modifications, none of these mechanisms are mutually exclusive. p300 is known to bind to DNA-binding transcription factors, general transcription factors (GTFs), and coactivators implicated in gene bookmarking, including FOXA1 [9], GATA1 [8], MLL [13], RUNX2 [65], TBP [4], [7,7], and Brd4 [12] [51]. Therefore, the maintenance of transcriptional memory by these factors is likely to involve p300 and other components of stabilized PIC assemblies. Though the overwhelming majority of both pol II and p300 are displaced from chromatin during mitosis, small populations of each have been detected within mitotic chromatin in prior studies depending on the cell type analyzed [38], [59]. In the presence of p300, the pre-initiation complex forms an assembly that is stabilized by multiple overlapping and synergistic interactions between different DNA:protein and protein:protein interfaces. Notably, p300 interacts with the TFIID complexes through two separate domains and forms a complex with phospho-CREB via its KID domain while maintaining interactions with pol II through its C-terminal domain [66]–[69]. Also CREB, in addition to contacting p300, interacts with TFIID via its Q2 domain [70]. Both p300 and TFIID (through its TAF1 component) bind to acetylated histone via their bromodomains, an interaction that is synergistically self-reinforced by their intrinsic HAT activity [71]. The observation that p300 deficiency decreases the level of TBP bound to the *FOS* promoter during mitosis suggests that p300 scaffolding and HAT activity play a central role in PIC stabilization at these genes by providing both an increased common interface for protein interactions and by favoring TBP:DNA interactions through local disruption of the repressive nucleosome structure via histone acetylation to increase accessibility. Other mechanisms to increase p300 concentration at the G2 phase may include the targeting of p300 to locally enriched concentrations of PCNA at chromosomal replication forks in S-phase through the known interactions between p300 and PCNA [72].

The precise role for pol II in bookmarked PIC complexes containing p300 remains to be fully defined, however the fact that the initiated and engaged polymerase makes extensive contacts with DNA, suggest that pol II could play a major stabilizing function in the bookmarked complex [73]. Recent descriptions of dynamic bookmarking of the *FOS* promoter by p300 and pol II complexes suggest that a significant portion of pol II in these complexes is unengaged or at least is in a dynamic equilibrium with engaged complexes [20]. It is therefore quite possible that the complexes that reassemble at the *FOS* promoter following passage of the replication fork and those that persist amongst condensed mitotic chromatin may undergo a lower rate of initiation and elongation until later stages of mitosis when more post-recruitment factors, like Brd4, become available [12], [55], [60]. This is consistent with the observations that heterochromatic or silenced chromatin may still be permissive to PIC assembly [74]. In addition, recent findings that pol II containing complexes are effective barriers to progression of the replication fork [75] and may act as insulators [76] provide additional examples of expanding roles for pol II containing complexes in the epigenetic transmission of remembered states of gene control.

Finally, a commonly described characteristic attributed to epigenetic inheritance is the stable passage to cellular progeny, through a mechanism that perpetuates or “renews” the epigenetic mark. The combined synergy between the bromodomains and HAT activity of p300 and TAF1 provides one means of auto-amplification. This is similar to what has been proposed for the chromodomains of the polycomb repressive complexes I (PRC1) subunits CBX4-8, and the EED subunits of polycomb repressive complexes 2 (PRC2), which bind to H3K27Me3 modifications to facilitate the recruitment of DNA methyltransferases (PRC1) and other associated chromatin modifiers (PRC1) and the EZH2 H3K27 histone methyltransferases (PRC2) respectively [77], [78]. It will be interesting to see if these self-perpetuating mechanisms may have a similar application in other molecular strategies for molecular retention of biochemically encoded information including cognitive memory [79]–[81].

## Materials and Methods

### Cell culture and cell proliferation

Jurkat T cells, HCT116-p300 WT and HCT116-derived p300 KO cells were treated with PMA (50 ng/ml) and Ionomycin (1 µM) (P/I) for 1 h. Then the cells were washed three times with medium supplemented with 10% FCS, resuspended in fresh medium and allowed to progress at 37°C with 5% CO_2_ incubator. Cell proliferation was carried out with CellTrace CFSE (carboxyfluorescein diacetate succinimidyl ester) (Molecular Probes) as recommended by the manufacturer. Briefly, CFSE was dissolved at 5 mM in DMSO prior to use and added to a final concentration of 1.5 µM. Analysis was performed on 100,000 cells using the FACSCalibur and Cell Quest Pro Software (BD Biosciences). Centrifugal elutriation was carried out as previously described [24].

### p300 KO cells

HCT116-p300 WT, HCT116-derived p300 KO, MEF-derived p300 WT and MEF-derived p300 −/− used were as described [20], [56].

HEK293LTV (Cell Biolabs, Inc) cells are transfected with Lipofectamine 2000 (Invitrogen) following manufacture's procedure, with a ratio of 20:15:6 packaged plasmid (GIPZ Lentivirus shRNA (Thermo Scientific), psPAX2 and pMD2G. After 24 h, viral supernatant is harvested, spin down and filter to remove cell debris (0.45 µm PVDF, Millipore). For HEK293 cells, 8 µg/ml polybrene (Sigma) is added to the viral supernatant and placed on cells. The viral supernatant harvest and subsequent placement is repeated for a total of three times (72 h of transduction with fresh viral supernatant at each 24 h period). For Jurkat T-cells, viral supernatant with 8 µg/ml polybrene (Sigma) were transduced by spinoculation at 32°C at 1200×g for 2 h. Cells were split as necessary to maintain log phase and 72 h post-transduction, cells were subjected to selection in 0.5 µg/ml (HEK293) or 0.25 µg/ml (Jurkat) puromycin (InvivoGen). Selection was maintained for one week. HEK293 cells were sorted on a FACSAria II (Becton Dickinson), based on the top 1/3 of the GFP output. The proliferations of transduced cells were carried out in the presence of puromycin.

### Flow cytometric analysis

Cells were collected, washed with ice-cold PBS, resuspended in 500 µl PBS and fixed in 5 ml ice-cold 70% ethanol. The cells were stored in −20°C until analysis where they were washed with 5 ml ice-cold PBS, permeabilized with 100 µl of 0.1% Triton-X and stained with 50 µg/ml Propidium Iodide and 300 µg/ml RNaseA (BD Pharmingen & Sigma). Analysis was performed on 10,000 cells using the FACS Calibur and Cell Quest Pro Software (BD Biosciences) with gating to eliminate cell aggregates and debris. For the transfected cell, the cells were fixed with 1% paraformaldehyde/PBS instead of 70% ethanol.

### Chromatin Immunoprecipitation (ChIP) and gene expression analysis

For ChIP assay, cells were fixed in formaldehyde and carried out as previously described [20]. Total RNA and reverse transcription reactions were prepared using RNAeasy and QuantiTect Reverse Transcription kits (Qiagen) according to the manufacturer's protocol.

#### ChIP-seq data analysis

The 36-mer short-read tags were mapped to the UCSC Genome Browser (Feb 2009, GRCh37/hg19). Detailed ChIP-seq data analysis was performed as previously described [82].

### Cell cycle synchronization

Cells were arrested in metaphase (M-phase) with nocodazole (Sigma). For Jurkat T-cells, 400 ml of 5×10^5^ cells/ml were treated with nocodazole at 400 ng/ml for 24 h. Cells were washed with PBS with nocodazole and resuspended in 10 ml PBS with nocodazole. Viable cells and debris were separated by centrifugation through the LSM Lymphocyte Separation Medium (ICN Biomedicals, Inc.) by layering in each 15 ml conical tube with 5 ml cell suspension to 4 ml LSM Lymphocyte Separation Medium. Centrifugation was carried out at 400×g for 30 min at room temperature. The lymphocyte layer was then washed with 15 ml PBS with nocodazole once and 15 ml PBS by centrifugation at 160×g for 5 min at room temperature. Cell pellet was resuspended in 20 ml PBS; 2 ml was used for cell cycle analysis by flow cytometric, 1 ml for RNA extraction and the remainder was used for ChIP assay. The p300 WT and p300 KO cells were treated with nocodazole at 100 ng/ml for 16 h. Then the cells were washed three times with medium supplemented with 10% FCS, resuspended in fresh medium and allowed to progress through the cell cycle at 37°C with 5% CO_2_ incubator. Cells were collected at indicated time point and stained for flow cytometric analysis, RNA extraction and ChIP assay.

### Chromosome conformation capture (3C)

The 3C assay was performed as described previously [83] with the modification that digestions was performed with EcoR1 restriction enzyme (New England Biolabs).

## Supporting Information

Figure S1 **Parental trans-generational transmision of remembered states.**
(PDF)

Figure S2 **Cell cycle phase specific purification of Jurkat T-cells by centrifugal elutriation.**
(PDF)

Figure S3 **Activating chromatin marks persist at the** ***FOS*** **across the cell cycle.**
(PDF)

Figure S4 **Stable retention of Pol II and p300 at promoters in mitotic chromatin is gene specific.**
(PDF)

Figure S5 **Subpopulations of p300 are retained on mitotic chromatin.**
(PDF)

Figure S6 **Nocodazole produces highly enriched populations of cells in M-phase.**
(PDF)

Figure S7 **Both p300 depleted and CREB inhibited cells show delayed entry into G1.**
(PDF)

Figure S8 **Confirmation of p300 depletion in HEK293 cells.**
(PDF)

Figure S9 **Promoter histone variant deposition and H3K4Me3 activating modifications are p300 independent in mitotic chromatin.**
(PDF)

Figure S10 **Multiple histone modifications do not appear quantitatively altered in p300 depleted cells.**
(PDF)

File S1 **p300 ChIP-Seq enhancer peaks.**
(XLS)

Methods S1 **Materials and Methods.**
(DOC)
