# Correction: Promoter-Bound p300 Complexes Facilitate Post-Mitotic Transmission of Transcriptional Memory

**DOI:** 10.1371/journal.pone.0106126

**Published:** 2014-08-18

**Authors:** The PLOS ONE Staff

## Notice of Republication

This article was republished on July 28th, 2014, to replace an erroneously published version. The publisher apologizes for this error. Please download this article again to view the corrected article. The originally published, uncorrected article and the republished, corrected article are provided here for reference.

## Supporting Information

File S1 Originally published, uncorrected article
(PDF)

File S2 Republished, corrected article
(PDF)
