# Supplementary material for: Promoter-Bound p300 Complexes Facilitate Post-Mitotic Transmission of Transcriptional Memory
Source: PLoS One. 2014 Jun 19;9(6):e99989. doi: 10.1371/journal.pone.0099989 (PMC4063784; doi:10.1371/journal.pone.0099989)
Supplement: Figure S1 — Parental trans-generational transmision of remembered states. (PDF) [file pone.0099989.s001.pdf]

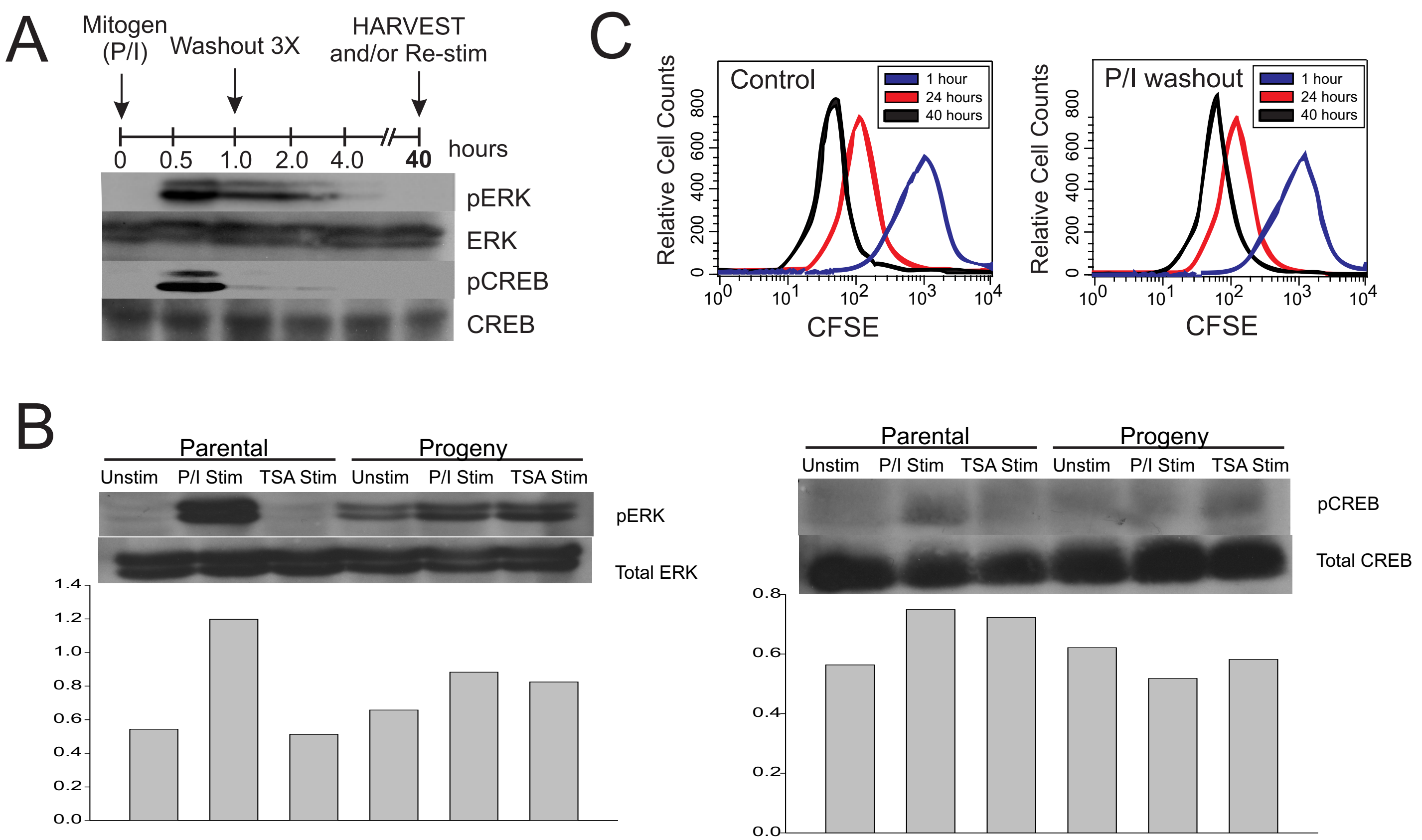

Supplementary Figure S1. Parental trans-generational transmision of remembered states. (A) Schematic of the experimental design. Jurkat cells were treated with PMA and Ionomycin (P/I) for 1 h. Cells were then washed and allowed to rest for 40 h. Western blots showing protein level of phospho-(Thr202/Tyr204) ERK, Total ERK, phospho- (Ser133) CREB and Total CREB at the respective time point. Shown is 1 of 2 independent biological replicate. (B) Resting Jurkat cells ("parental") or pre-treated with P/I (mitogen pulsed) for 1 h, washed, allowed to rest for 40 h ("progeny") and restimulated with P/I (30 min) and TSA (2 h). Western blots showing protein level of phospho-(Thr202/Tyr204) ERK, Total ERK, phospho- (Ser133) CREB and Total CREB. Shown is 1 of 2 independent biological replicate (C) Flow cytometric (FACS) analysis of CFSE with Jurkat cells comparing control and P/I washout cells to indicate cell proliferation or cell division..

Fig S1
