# Supplementary material for: Promoter-Bound p300 Complexes Facilitate Post-Mitotic Transmission of Transcriptional Memory
Source: PLoS One. 2014 Jun 19;9(6):e99989. doi: 10.1371/journal.pone.0099989 (PMC4063784; doi:10.1371/journal.pone.0099989)
Supplement: Figure S2 — Cell cycle phase specific purification of Jurkat T-cells by centrifugal elutriation. (PDF) [file pone.0099989.s002.pdf]

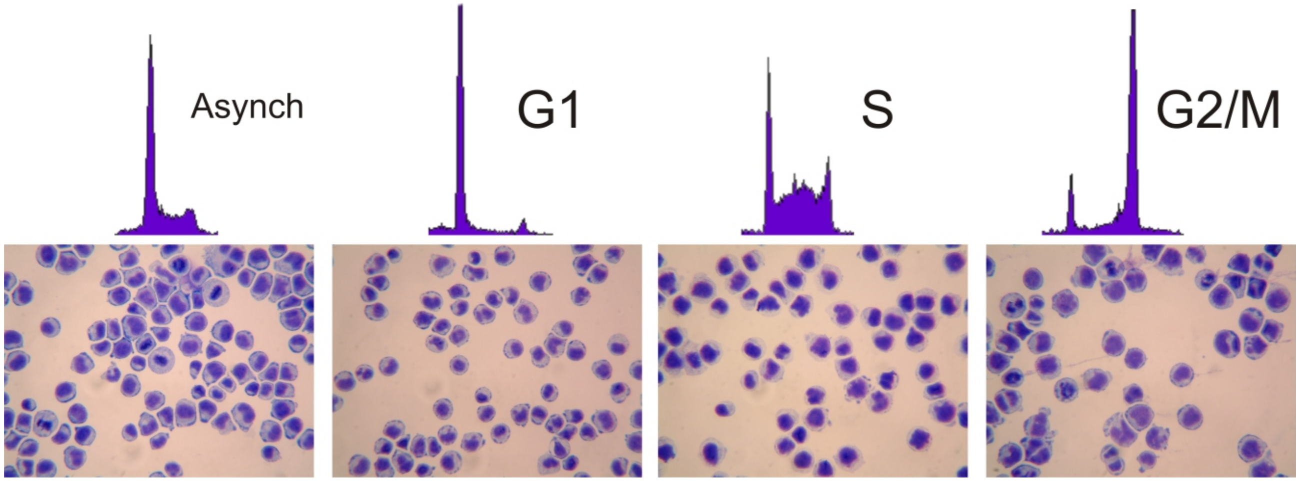

	Asynch	G1	S	G2/M
G0/G1 (%)	68.09	90.71	16.79	8.33
S (%)	18.27	1.80	73.80	13.88
G2/M (%)	12.22	1.92	9.41	77.80

Supplementary Figure S2. Cell cycle phase specific purification of Jurkat T-cells by centrifugal elutriation. Jurkat cells were elutriated to obtain purified populations of cells at different stages of the cell cycle. (Top) Cell cycle analysis by flow cytometric (FACS) analysis and (Bottom) Wright's and Giemsa staining of centrifugal elutriated Jurkat cells.

Fig S2
