# Supplementary material for: Promoter-Bound p300 Complexes Facilitate Post-Mitotic Transmission of Transcriptional Memory
Source: PLoS One. 2014 Jun 19;9(6):e99989. doi: 10.1371/journal.pone.0099989 (PMC4063784; doi:10.1371/journal.pone.0099989)
Supplement: Figure S3 — Activating chromatin marks persist at the FOS across the cell cycle. (PDF) [file pone.0099989.s003.pdf]

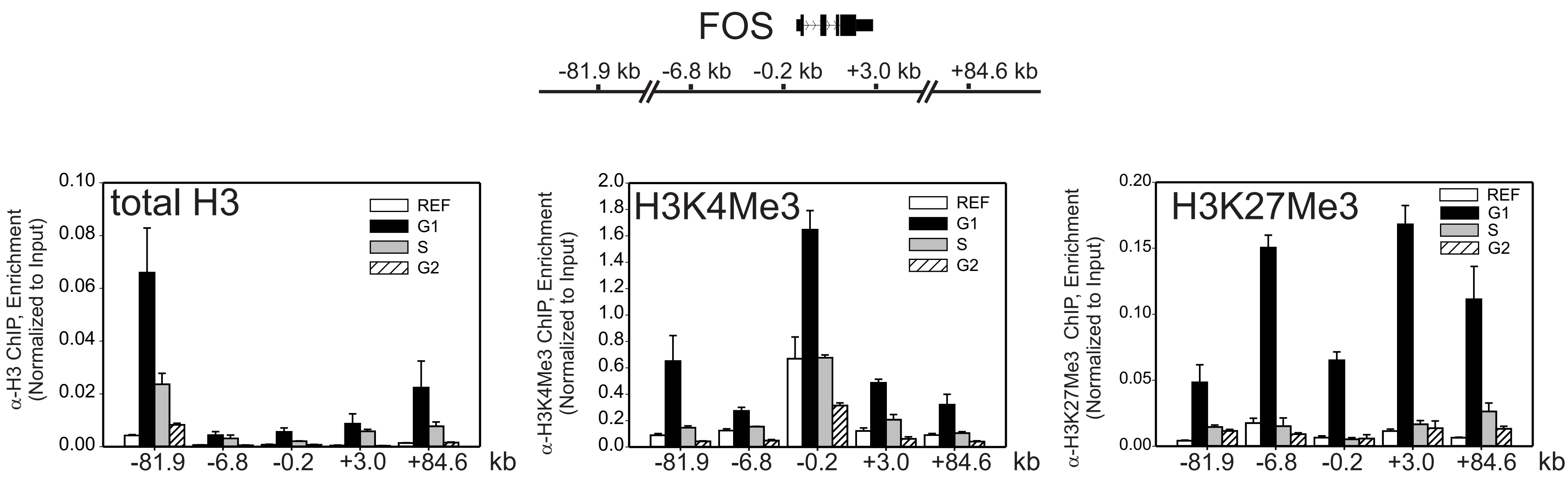

Supplementary Figure S3. Activating chromatin marks persist at the *FOS* across the cell cycle. Jurkat cells were elutriated to obtain populations of cells at different stages of the cell cycle. A schematic of the locations of upstream (-81.9 kb & -6.8 kb), promoter (-0.2 kb), last exon (+3.0 kb) and downstream (+84.6 kb) at the *FOS* locus relative to TSS. Position dependent profile at the *FOS* locus for total Histone H3, H3 Lys 4 tri-methylation (H3K4Me3) and H3 Lys 27 tri-methylation (H3K27Me3) determined by quantitative ChIP analysis. Error bars represent standard error of mean from 2 or 3 biological replicates each determined in duplicate.

Fig S3
