# Supplementary material for: Promoter-Bound p300 Complexes Facilitate Post-Mitotic Transmission of Transcriptional Memory
Source: PLoS One. 2014 Jun 19;9(6):e99989. doi: 10.1371/journal.pone.0099989 (PMC4063784; doi:10.1371/journal.pone.0099989)
Supplement: Figure S4 — Stable retention of Pol II and p300 at promoters in mitotic chromatin is gene specific. (PDF) [file pone.0099989.s004.pdf]

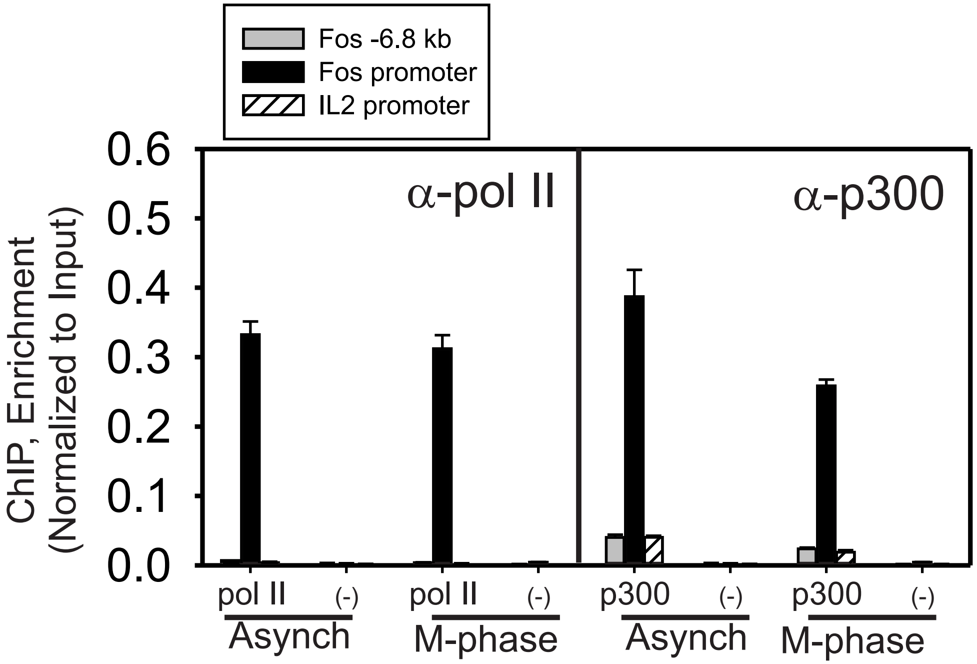

Supplementary Figure S4. Stable retention of Pol II and p300 at promoters in mitotic chromatin is gene specific. Jurkat cells were treated with nocodazole (400 ng/ml) for 24 h to obtain metaphase (M-phase) population. Recruitment of Pol II, p300 and no antibody control (-) on the *FOS* -6.8 kb, *FOS* promoter and IL2 promoter as indicated determined by quantitative ChIP analysis. Error bars represent standard error of mean from 2 biological replicates each determined in duplicate.
