# Supplementary material for: Promoter-Bound p300 Complexes Facilitate Post-Mitotic Transmission of Transcriptional Memory
Source: PLoS One. 2014 Jun 19;9(6):e99989. doi: 10.1371/journal.pone.0099989 (PMC4063784; doi:10.1371/journal.pone.0099989)
Supplement: Figure S5 — Subpopulations of p300 are retained on mitotic chromatin. (PDF) [file pone.0099989.s005.pdf]

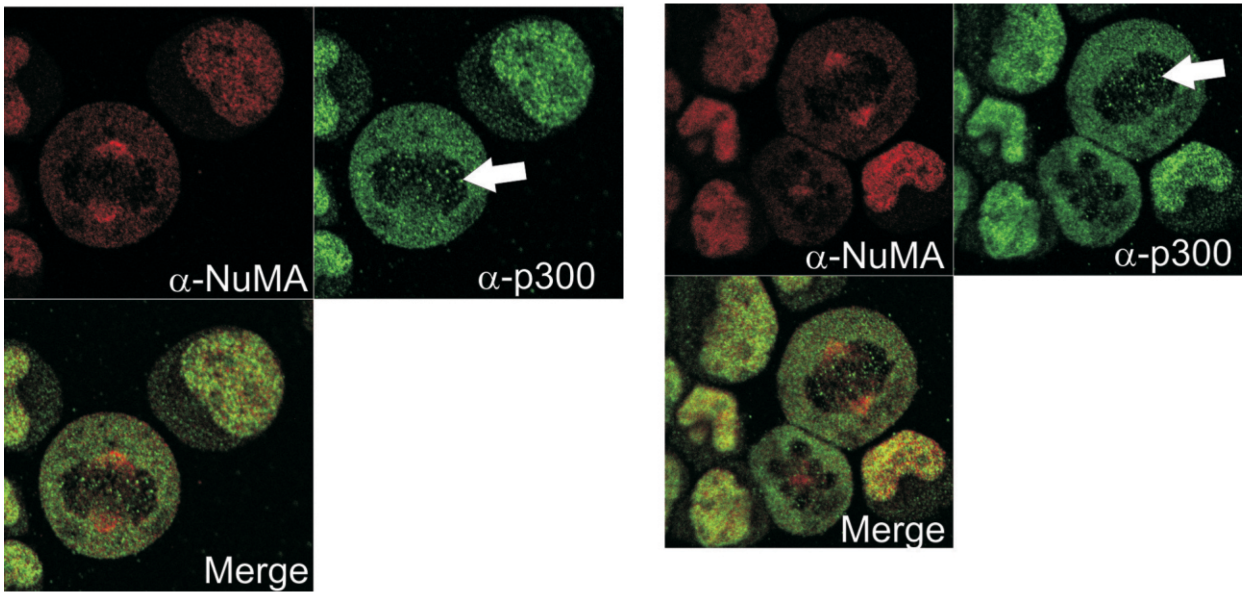

Supplementary Figure S5. Subpopulations of p300 are retained on mitotic chromatin. Fixed Jurkat cells were doubled labeled with α-nuclear mitotic apparatus protein (NuMA) (as detected with Alexa Fluor® 594 anti-mouse antibody, red) and α-p300 (as detected with Alexa Fluor® 488 anti-rabbit antibody, green) antibodies. The arrow indicates the presence of p300 in chromatin of a mitotic cell.

Fig S5
