# Supplementary material for: Promoter-Bound p300 Complexes Facilitate Post-Mitotic Transmission of Transcriptional Memory
Source: PLoS One. 2014 Jun 19;9(6):e99989. doi: 10.1371/journal.pone.0099989 (PMC4063784; doi:10.1371/journal.pone.0099989)
Supplement: Figure S6 — Nocodazole produces highly enriched populations of cells in M-phase. (PDF) [file pone.0099989.s006.pdf]

Jurkat T-cells

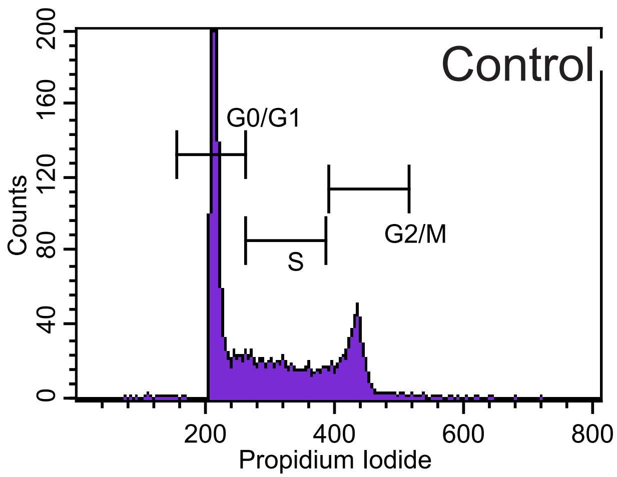

Marker	% Gated	Mean
All	100.00	281.71
G0/G1	57.53	218.74
S	23.43	319.09
G2/M	17.90	426.10

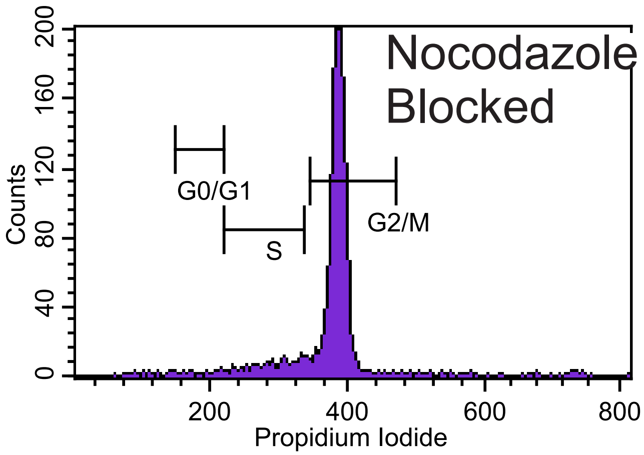

Marker	% Gated	Mean
All	100.00	375.37
G0/G1	0.94	188.06
S	7.81	292.63
G2/M	88.15	383.65

Supplementary Figure S6. Nocodazole produces highly enriched populations of cells in M-phase. FACS analysis of Jurkat cells treated with nocodazole (400 ng/ml) for 24 h to obtain metaphase (M-phase) population.

Fig S6
