# Supplementary material for: Promoter-Bound p300 Complexes Facilitate Post-Mitotic Transmission of Transcriptional Memory
Source: PLoS One. 2014 Jun 19;9(6):e99989. doi: 10.1371/journal.pone.0099989 (PMC4063784; doi:10.1371/journal.pone.0099989)
Supplement: Figure S7 — Both p300 depleted and CREB inhibited cells show delayed entry into G1. (PDF) [file pone.0099989.s007.pdf]

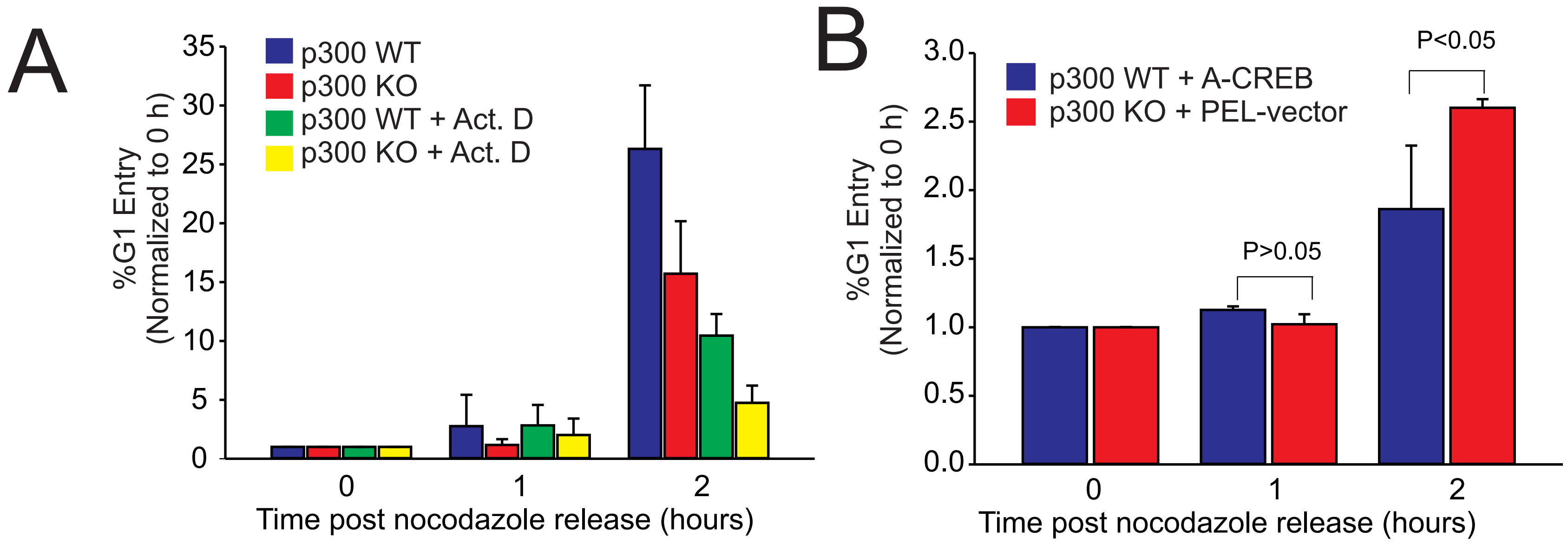

Supplementary Figure S7. Both p300 depleted and CREB inhibited cells show delayed entry into G1. p300 WT and p300 KO cells were treated as in Figure 3B. A graphical representation of G1 cells analyzed by FACS comparing p300 WT and p300 KO cells (A) in the presence of the transcriptional inhibitor actinomycin D or (B) cells expressing dominant negative CREB upon nocodazole treatment and released.

Fig S7
