# Supplementary material for: Promoter-Bound p300 Complexes Facilitate Post-Mitotic Transmission of Transcriptional Memory
Source: PLoS One. 2014 Jun 19;9(6):e99989. doi: 10.1371/journal.pone.0099989 (PMC4063784; doi:10.1371/journal.pone.0099989)
Supplement: Figure S8 — Confirmation of p300 depletion in HEK293 cells. (PDF) [file pone.0099989.s008.pdf]

A

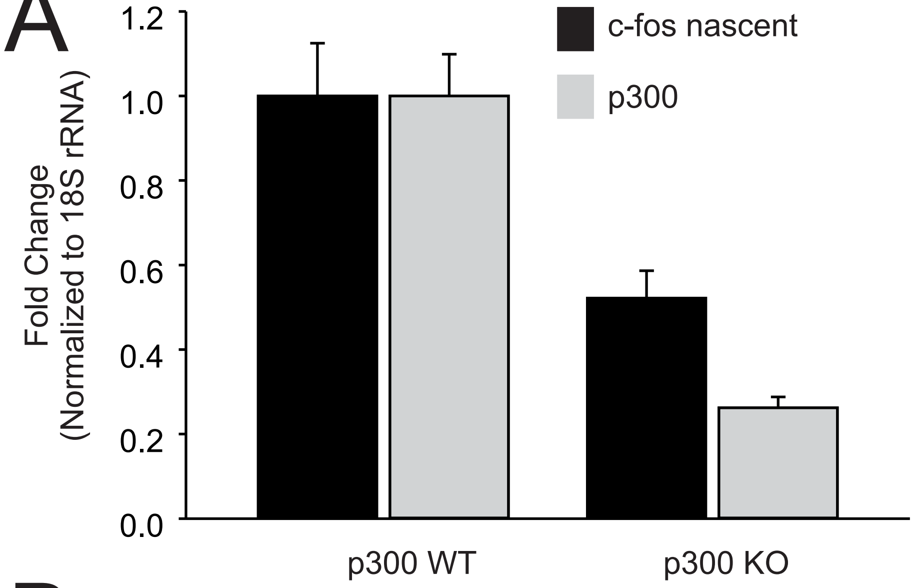

B

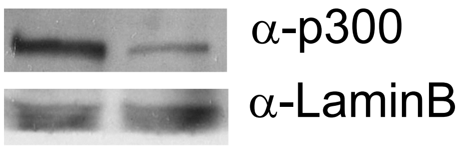

Supplementary Figure S8. Confirmation of p300 depletion in HEK293 cells. (A) qRT-PCR profile showing expression of *FOS* and p300 respectively of p300 WT and p300 KO in HEK293 cell. (B) Western blot showing protein level of p300 and LaminB as loading control.

Fig S8
