# Supplementary material for: Promoter-Bound p300 Complexes Facilitate Post-Mitotic Transmission of Transcriptional Memory
Source: PLoS One. 2014 Jun 19;9(6):e99989. doi: 10.1371/journal.pone.0099989 (PMC4063784; doi:10.1371/journal.pone.0099989)
Supplement: Figure S9 — Promoter histone variant deposition and H3K4Me3 activating modifications are p300 independent in mitotic chromatin. (PDF) [file pone.0099989.s009.pdf]

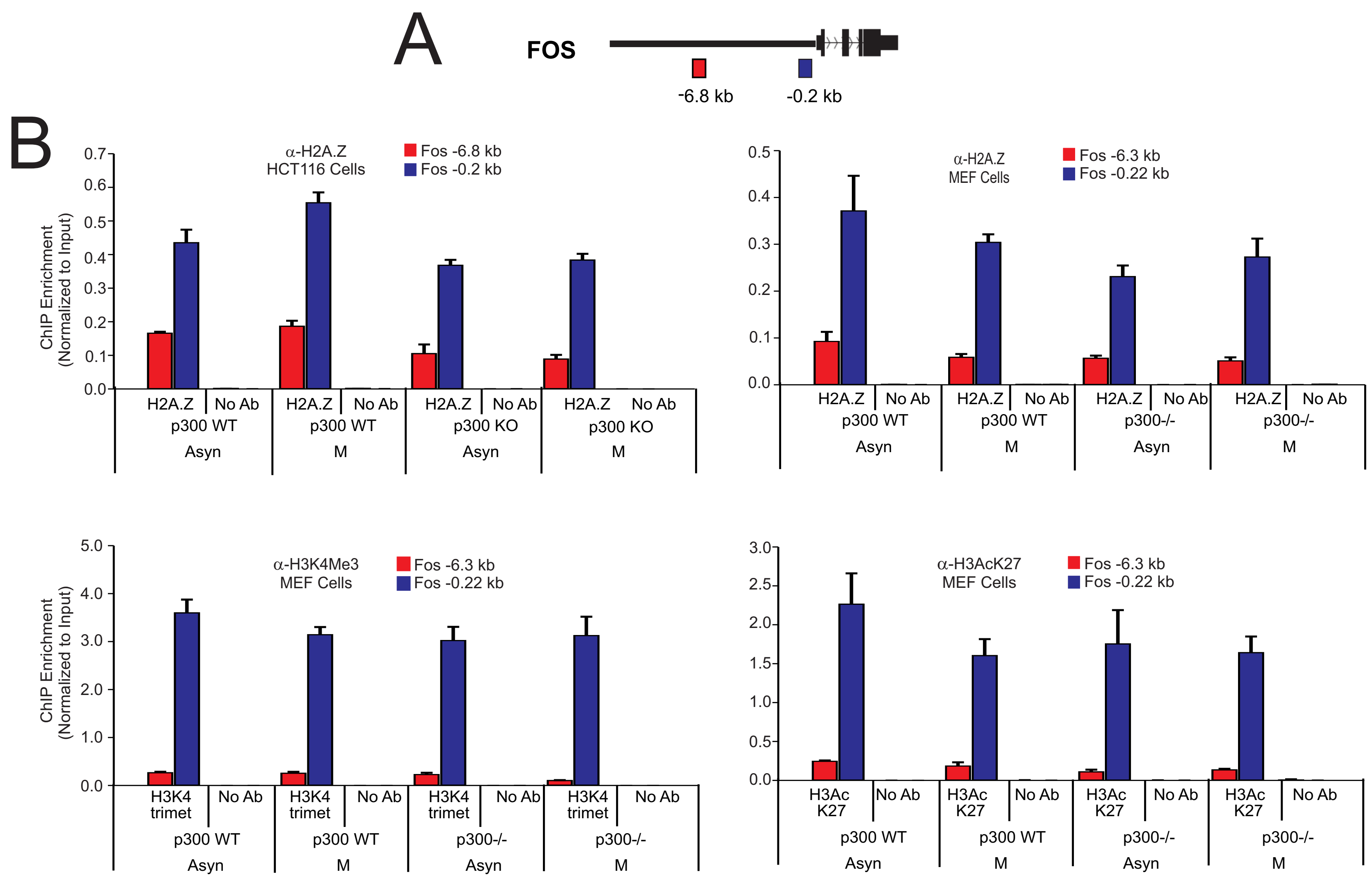

Supplementary Figure S9. Promoter histone variant deposition and H3K4Me3 activating modifications are p300 independent in mitotic chromatin. (A) A schematic of the locations of upstream (-6.8 kb) and promoter (-0.2 kb) at the *FOS* locus relative to TSS. (B) p300 WT and p300 KO cells were treated with nocodazole (100 ng/ml) for 16 h to obtain metaphase (M-phase) population. Position dependent profile at the *FOS* locus at indicated position (relative to TSS) for indicated antibodies and no antibody control as indicated determined by quantitative ChIP analysis. Error bars represent standard error of mean from 2 biological replicates each determined in duplicate.

Fig S9
