# Supplementary material for: Promoter-Bound p300 Complexes Facilitate Post-Mitotic Transmission of Transcriptional Memory
Source: PLoS One. 2014 Jun 19;9(6):e99989. doi: 10.1371/journal.pone.0099989 (PMC4063784; doi:10.1371/journal.pone.0099989)
Supplement: Figure S10 — Multiple histone modifications do not appear quantitatively altered in p300 depleted cells. (PDF) [file pone.0099989.s010.pdf]

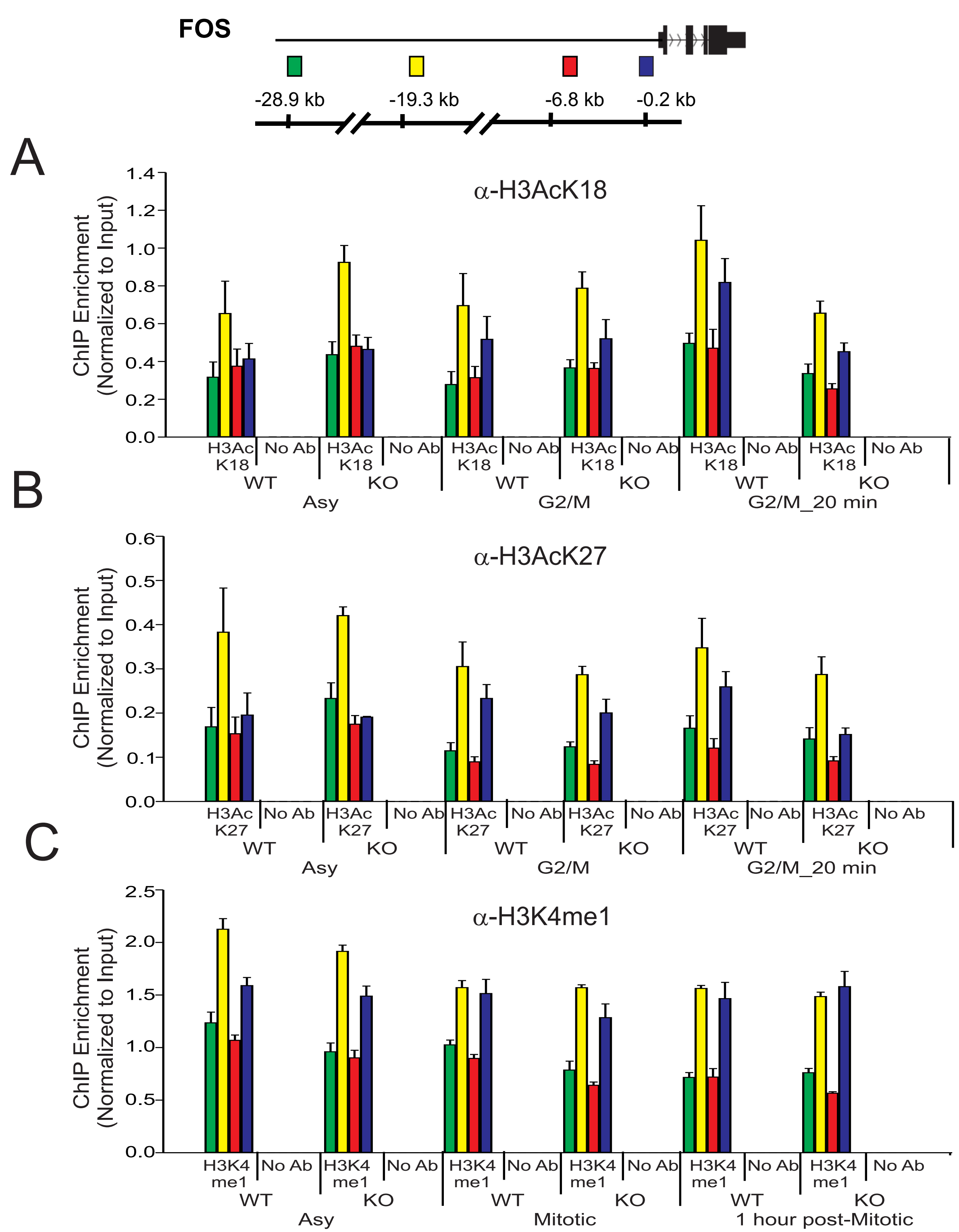

Supplementary Figure S10. Multiple histone modifications do not appear quantitatively altered in p300 depleted cells. A schematic of the locations of enhancers (-28.9 kb & -19.3 kb), upstream (-6.8 kb) and promoter (-0.2 kb) at the *FOS* locus relative to TSS. Jurkat p300 WT and p300 KO cells were elutriated to obtain cells of G2/M population and allowed to progress for 20 min to the G1 phase of the cell cycle. Position dependent profile at the *FOS* locus for (A) H3 Lys 18 acetylation (H3K18ac) and (B) H3 Lys 27 acetylation (H3K27ac) and no antibody control as indicated determined by quantitative ChIP analysis. Error bars represent standard error of mean from 2 biological replicates each determined in duplicate. (C) p300 WT and p300 KO in HCT 116 cells were treated as Figure 3B, and released for 1 h post-mitotic. Position dependent profile at the *FOS* locus for H3 Lys mono-methylation (H3K4Me1) and no antibody control as indicated determined by quantitative ChIP analysis. Error bars represent standard error of mean from 2 biological replicates each determined in duplicate.

Fig S10
