# Supplementary material for: Promoter-Bound p300 Complexes Facilitate Post-Mitotic Transmission of Transcriptional Memory
Source: PLoS One. 2014 Jun 19;9(6):e99989. doi: 10.1371/journal.pone.0099989 (PMC4063784; doi:10.1371/journal.pone.0099989)
Supplement: Methods S1 — Materials and Methods. (DOC) [file pone.0099989.s012.doc]

**Supplementary Materials Methods:**

*Cell culture and cell proliferation*

All cell lines were maintained at 37°C with 5%CO_2_ in a humidified atmosphere. Jurkat T and HEK293 cells were cultured in RPMI medium (Gemini) supplemented with 10% FBS and 100 U/ml penicillin and streptomycin. HCT116 cells were maintained in MCCoy’s 5A (Mediatech) supplemented with 10% FBS and 100 U/ml penicillin and streptomycin. MEF cells were maintained in DMEM (Hyclone) supplemented with 20% FBS and 100 U/ml penicillin and streptomycin.

*Microscopy and immuno-fluorescence (IF) analysis*

Centrifugation elutriated Jurkat cells at 5 x 10^4^ in 100 µl media were cytocentrifuged (Cytospin 3 Cytocentrifuge, Thermo Scientific) on a Poly-L-Lysine (Sigma) coated slide (Daigger). Wright’s and Giemsa staining were carried out with Shandon Kwik-Diff™ Stains (Thermo Scientific) as recommended by the manufacturer. Immuno-fluorescence (IF) with 1:50 dilution of p300/ 1:400 dilution of Alexa Fluor® 488 goat anti-rabbit IgG (Invitrogen) and 1:50 dilution nuclear mitotic apparatus protein (NuMA) (BD Biosciences)/ 1:400 dilution of Alexa Fluor® 594 goat anti-mouse IgM (Invitrogen) antibodies. Fluorescent and bright field images were captured on Zeiss Axiovert S-100 inverted microscope using a 10X, 40X or 60X (NA 1.6, oil emmersion) lens. Images were processed on a Spot Camera CCD image analysis system utilizing the Spot Software v2.2 (Diagnostic Instruments Inc., Sterling Heights, MI). Confocal images were collected on a Zeiss Pascal 510 laser scanning microscope at magnifications of 40X and 100X and analyzed using the Zeiss LSM Image Examiner Software v2.5.

*ChIP-western*

For ChIP-western, same conditions as for ChIP were used. Beads were boiled for 10 mins in PAGE-loading dye containing 200mM β-mercaptoethanol to elute proteins. Samples were run on 6% Tris-glycine –SDS-PAGE and western blots were carried out with the indicated antibodies.

*Transfection*

p300 WT HCT 116 cells were transfected with a MCherry-tag-dominant-negative CREB (Butscher et al., 1998) and a GFP-tag plasmid with Lipofectamine2000 (Invitrogen) as recommended by the manufacturer. Twenty four hours post-transfection, the cells were synchronized as mentioned.

Primer sequences (RT-PCR):

| No. | Gene | Forward sequence | Reverse sequence |
| --- | --- | --- | --- |
| 1. | 18S rRNA | GCCCGAAGCGTTTACTTTGA | TCCATTATTCCTAGCTGCGGTATC |
| 2. | CDC6 | GGGAATCAGAGGCTCAGAAG | CACTGGATGTTTGCAGGAGA |
| 3. | CCNB1 | CAGATGTTTCCATTGGGCTT | GAACCTGAGCCAGAACCTGA |
| 4. | E2F-1 | GGCCAGGTACTGATGGTCA | GACCCTGACCTGCTGCTCT |
| 5. | CD69 | ATTTTGGGCTGAAGTCCAGC | TGGGCCAATACAATTGTCCAGG |
| 6. | *FOS* (-28.9 kb) | GCCCATATCATCAGCCACTT | CCACCCCACTTGGTTGTATC |
| 7. | *FOS* (-19.3 kb) | TCCTACCGATTACAGCCCAG | ATATGGAAAGGCCTCCGAGT |
| 8. | *FOS* (-81.9 kb) | CACTTCCTCTTTCCCCAGTG | TGCTCCAAGGTAAGACAGGG |
| 9.  10.  11.  12. | *FOS* (+84.6 kb)  **FOS* (-6.3 kb)  * *FOS* (-0.22 kb)  *FOS* eRNA | CCTCCAGTGGTTTCATCACC  tcaatgctttgaagcacgtc  ccccctaagatcccaaatgt  CCCTGACAATTATGGCCAAC | GTGGGGACTCAATAAGGGGT  cttgtgtggctctctggtca  gtcgcggttggagtagtagg  CAGGTCCTGAGCCAACATCT |

Additional primer sequences are as reported in Byun et al., 2009.

* Mouse locus

Primer sequences (3C):

| No. | Locus | Sequence |  |
| --- | --- | --- | --- |
| 1. | *FOS* (-26.6kb) | ACCCGGATGAGATAATGTGC |  |
| 2. | *FOS* (-23.8 kb) | AAATGCCCTTCCCACCTACT |  |
| 3. | *FOS* (-10.1 kb) | TTTGCAGCGTCCATACAAAG |  |
| 4. | *FOS* (-6.21 kb) | CCCGAGGTGGGAGAGTAGAC |  |
| 5. | *FOS* (-3.59 kb) | CCACAGGGAGAGTGCAAAGT |  |
| 6. | *FOS* (+6.0 kb) | TCCTCTCCTGGTCCCTGTTA |  |
| 7. | HBB (-4.18 kb) | ATGTCCCATCCAGGTGATGT |  |
| 8. | HBB (+5.07 kb) | GTGGGTGCAGGACAGTAGGT |  |

Antibodies:

| Protein | Source | Catalog no. |
| --- | --- | --- |
| pol II & p300 | Byun et al., 2009 |  |
| Brd4 | Jang et al., 2005 &  Bethyl Lab. A301-985A | |
| Brg1 | Wurster & Pazin, 2008 | |
| Cohesin (SMC1) | Bethyl Lab. | A300-055A |
| p-Cohesin (p-SMC1,S966) | Bethyl Lab. | A300-050A |
| CREB | Santa Cruz  Cell Signaling | sc-186  9197 |
| p-CREB (Ser133) | Millipore  Cell Signaling | 06-519  9191 |
| ELK-1 | Santa Cruz | sc-355 |
| p- ELK-1 (Ser383) | Santa Cruz | sc-8406 |
| p-ERK (phospho-p44/42 MAPK (Erk1/2)  (Thr202/Tyr204) | Cell Signaling | 4370 |
| c-*FOS* | Santa Cruz | sc-52 |
| H3 Lys 9,14 acetylation  (acetylated histone H3) | Millipore | 06-599 |
| H3 Lys 18 acetylation (H3K18ac) | Abcam | ab1191 |
| H3 Lys 27 acetylation (H3K27ac)) | Abcam | ab4729 |
| H4 Lys 5, 8, 12, 16 acetylation  (acetylated histone H4) | Millipore | 06-598 |
| Histone H2A.Z | Abcam | ab4174 |
| Histone H3 | Millipore | 06-755 |
| H3 Lys 4 mono-methylation (H3K4Me1) | Abcam | ab 8895 |
| H3 Lys 4 tri-methylation (H3K4Me3) | Millipore | 07-473 |
| H3 Lys 27 tri-methylation (H3K27Me3) | Millipore | 07-449 |
| LaminB | Santa Cruz | sc-6217 |
| MED1 (TRAP220) | Santa Cruz | sc-8998 |
| MED17 | Santa Cruz | sc-12453 |
| NuMA | BD Biosciences | 610561 |
| TBP (TFIID) | Santa Cruz | sc-273 |
| TORC2 | Calbiochem | ST1099 |
|  |  |  |

**References:**

**Byun,J.S., Wong,M.M., Cui,W., Idelman,G., Li,Q., De,S.A., Bilke,S., Haggerty,C.M., Player,A., Wang,Y.H., Thirman,M.J., Kaberlein,J.J., Petrovas,C., Koup,R.A., Longo,D., Ozato,K., and Gardner,K. (2009). Dynamic bookmarking of primary response genes by p300 and RNA polymerase II complexes. Proc. Natl. Acad. Sci. U. S. A. *106*, 19286-19291.**

**Jang,M.K., Mochizuki,K., Zhou,M., Jeong,H.S., Brady,J.N., and Ozato,K. (2005) The bromodomain protein Brd4 is a positive regulatory component**

**of P-TEFb and stimulates RNA polymerase II-dependent transcription. Mol Cell 19, 523–534.**

**Wurster,A.L. and Pazin,M.J. (2008). BRG1-mediated chromatin remodeling regulates differentiation and gene expression of T helper cells. Mol. Cell Biol. *28*, 7274-7285.**
